# Promoting a foundation of resilience in older adults: pilot trial of a strengths-based positive psychology intervention for chronic low back pain

**DOI:** 10.1080/21642850.2024.2434711

**Published:** 2024-12-05

**Authors:** Emily J. Bartley, Ben L. Ofri, Terrie Vasilopoulos, Shreela Palit, Calia A. Torres, Kimberly T. Sibille

**Affiliations:** a College of Dentistry, University of Florida, Gainesville, FL, USA; b College of Medicine, University of Florida, Gainesville, FL, USA; c Center for Healthcare Delivery Science, Nemours Children’s Health, Jacksonville, FL, USA; d Division of Hematology and Oncology, The University of Alabama at Birmingham, Birmingham, AL, USA

**Keywords:** Low back pain, resilience, positive psychology, intervention, aging

## Abstract

**Introduction:**

Chronic low back pain (cLBP) is a global public health problem and a leading cause of disability among older adults. Recent years have seen a burgeoning interest in promoting resilience in individuals with chronic pain; however, therapeutic strategies that focus on positive psychological resources and individual strengths are understudied among older adult populations. The aim of this study was to examine the feasibility and acceptability of a resilience-promoting intervention among older adults with cLBP, and to assess preliminary treatment effects on pain severity, pain interference, and quality of life.

**Methods:**

Participants included 50 adults, ages ≥50 years, with back pain rated (at minimum) of moderate intensity and having a significant impact on daily activities. This single-arm trial included seven weekly group sessions targeting positive psychology concepts and techniques that have established benefits in pain management.

**Results:**

Results suggest high credibility and engagement in the intervention program. Participants found the weekly session content to be beneficial and global treatment satisfaction was high. Pain intensity (−4.3 [−6.14, −2.54]), pain interference (−3.3 [−4.83, −1.80]), depression (−1.6 [−3.16, −0.04]) and QoL-health satisfaction (0.3 [0.01, 0.55]) improved from pre- to post-intervention. At the 3-month follow-up, improvements were also observed for pain intensity (−2.1 [−9.29, −5.04]), pain interference (−5.3 [−6.54, −2.86]), and QoL-physical health (0.9 [0.11, 1.74]).

**Discussion:**

Results support the feasibility, acceptability, and potential efficacy of a resilience intervention for older adults with cLBP. These findings may be a step toward the advancement of therapeutic pain modalities and provide a foundation for future research on resilience-promoting interventions for aging populations with chronic pain.

## Introduction

Chronic low back pain (cLBP) is a debilitating condition afflicting millions worldwide and is associated with significant decrements in physical and psychological functioning. It is one of the leading causes of disability among older adults (Wong et al., 2017; Wu et al., 2020), making it a principal source for healthcare utilization (St. Sauver et al., 2013). With the aging population growing, the prevalence of cLBP is expected to increase significantly (Freburger et al., 2009), rendering pain management among older adults a top health priority.

Traditionally, pain research has primarily focused on identifying vulnerability and risk factors (e.g. depression, anxiety) that negatively impact pain and overall functioning. However, this approach addresses only part of the broader picture as evidence suggests that individuals with chronic pain possess the capacity for resilience (Sturgeon & Zautra, 2010). Resilience, broadly defined, is a dynamic and multifaceted construct characterized by the capacity for recovery, growth, adaptation, and resistance to a challenge or stressor (Brown et al., 2023). In the context of pain, individuals with a greater degree of protective and promotive factors are known to adapt more effectively to disruptions in physiological, emotional, or cognitive functioning – whether in response to a pain flare-up or recovery from surgery – continue engaging in meaningful and valued activities, and experience personal growth through their experiences with chronic pain (Sturgeon & Zautra, 2010). While various risk factors heighten pain vulnerability, positive psychosocial factors – such as self-efficacy and positive emotions – are recognized for fostering adaptive pain-related functioning and enhancing overall quality of life, both of which are key indicators of resilience (Bartley et al., 2017; Sturgeon & Zautra, 2010).

A growing body of literature signifies the importance of positive affective states in promoting resiliency and optimal pain outcomes (Finan & Garland, 2015; Ong et al., 2020), including recent work in cLBP highlighting various protective factors (i.e. positive affect, gratitude, optimism) as strong predictors of lower clinical pain and disability, as well as enhanced psychological function and quality of life (Bartley et al., 2019; Bartley et al., 2023; Kreis et al. 2015; Makhoul & Bartley, 2023; Palit et al., 2020). This growing interest in the benefits of positive emotions has sparked an increasing focus on positive psychology interventions (PPIs) for chronic pain. Such interventions are strength-based and capitalize on an array of activities (e.g. gratitude expression, hopeful thinking) to foster positive emotions, cognitions, and behaviors and differ from psychological interventions that predominantly focus on the reduction of negative symptomatology (e.g. pain, negative affect). Aligning with the broaden-and-build theory of positive emotions, it is believed that PPIs enhance resilience and health-related functioning (e.g. pain, well-being) through the development of adaptive cognitions and approach-oriented behaviors that cultivate social, physical, and psychological resources (Fredrickson, 2004). Meta-analytic reviews in both clinical and non-clinical populations have observed beneficial effects of PPIs on negative cognitive and emotional states (i.e. anxiety, depression, pain catastrophizing), psychological and subjective well-being, and pain severity (Braunwalder et al., 2022; Hendriks et al., 2020). Further, multiple studies have supported the utility of PPIs by incorporating a variety of positive activities such as gratitude expression, savoring, kindness, personal strengths, and induced optimism across a range of pain conditions including osteoarthritis, physical disability, fibromyalgia, and musculoskeletal pain (Boselie et al., 2018; Flink et al., 2015; Hausmann et al., 2014; Hausmann et al., 2017; Hausmann et al., 2018; Janevic et al., 2022; Müller et al., 2016; Müller et al., 2022; Ong et al., 2023; Peters et al., 2017). Thus, therapeutic approaches aimed at fostering resilience through PPIs may be optimally positioned to promote successful aging and provide symptomatic improvement in individuals with chronic pain.

While the potential benefits of PPIs in chronic pain management are gaining recognition, there remains a significant gap in the existing literature, particularly concerning their application in cLBP and in older adults. Notably, only one study (involving a single case design of five participants) has targeted cLBP using activities such as self-compassion, three good things, best possible self, and savoring (Flink et al., 2015). Moreover, two randomized controlled trials (RCTs) using similar multicomponent activities examined the effects of PPIs in older adults, observing significant improvements in pain interference, pain self-efficacy, positive and negative affect, and pain catastrophizing relative to the control group (Janevic et al., 2022; Ong et al., 2023). While these findings are promising, further research is warranted to discern their feasibility and effectiveness in older adults with chronic pain. Given evidence signifying that older adults have increased adaptive emotion regulation capacities, yet experience decrements in eudaimonic well-being (i.e. sense of purpose, personal growth) with age, PPIs may have particular utility (Bar-Tur, 2021; Blanchard-Fields et al., 2004).

## Aims and hypotheses

The primary objective of this study was to assess the feasibility and acceptability of a group-based pilot trial titled, Adaptability and Resilience in Aging Adults (ARIAA), aimed at enhancing psychological resilience among older adults with cLBP. This intervention was grounded in positive psychological theory and incorporated components (e.g. promoting positive affect, gratitude, and hopeful thinking) and activities (e.g. mindfulness, activity pacing) with strong empirical evidence in chronic pain. The following aims and hypotheses were proposed:

### Aim 1 (Primary)

Evaluate the feasibility and acceptability of the intervention, as indicated by recruitment and retention metrics, home activity completion, session engagement, and treatment credibility and satisfaction.

### Aim 2 (Exploratory)

Examine preliminary intervention effects on back pain severity, pain interference, negative mood, and quality of life.

Our central hypothesis was that participants would have high retention and adherence to the intervention, including high rates of session engagement and satisfaction with the program. Furthermore, based on evidence that repeated experiences of positive emotions contribute to the development of psychosocial, cognitive, and physical resources that enhance psychological well-being and physical health (Fredrickson, 2001), we hypothesized that resilience would be bolstered through reductions in back pain severity and interference, negative mood, and improvements in quality of life from pre- to post-intervention.

## Materials and methods

### Study procedures

This trial included seven 1.5-h group intervention sessions scheduled once weekly for seven weeks. The full study protocol is described in ClinicalTrials.gov (Trial Registration: NCT04068922) and published elsewhere (Lysne et al., 2021). Recruitment for this single-arm clinical trial began in January 2020, with one in-person group (*n* = 7) completed prior to operations ceasing from the COVID-19 pandemic. Due to pandemic disruptions that impacted recruitment, the following protocol changes were made: (1) the group intervention was adapted from in-person sessions to remote delivery (via PHI Zoom), and (2) the recruitment age range was modified from ≥60 years of age to ≥50 years of age. Intervention sessions were transitioned back to in-person for the final group due to participant request (*n* = 6).

Ethics approval for all study procedures was provided by the University of Florida Institutional Review Board (IRB201901927) and all participants provided written informed consent prior to study procedures. All interested participants initially underwent a brief screening interview via telephone to determine eligibility. To ensure that no exclusion criteria were present, participants self-reported their age and health history including the presence of major medical illnesses, recent back-related injuries or surgeries, and low back pain symptoms. Participants were then invited to participate in a 1.5-h baseline assessment for a more thorough evaluation of eligibility.

During the baseline assessment, informed consent was reviewed, and participants then completed a demographic and medical history questionnaire followed by a series of psychological and pain-related questionnaires assessing study-relevant outcomes. As an evaluation of cognitive functioning, participants also completed the Montreal Cognitive Assessment (MoCA) (Nasreddine et al., 2005). Participants with scores <26 were excluded as this could interfere with their engagement in the study intervention. At the completion of the baseline assessment, eligible participants were scheduled for their first group intervention session. Outcomes were collected upon completion of the final group session. Participants also completed a treatment engagement questionnaire after each group session as a measure of treatment acceptability. Approximately one week after the final intervention session, participants engaged in a 20–30-minute follow-up interview with the study coordinator to provide context for the study findings and to gain feedback on their experience in ARIAA.

### Participant recruitment and eligibility

Individuals with cLBP were recruited via clinic and community-based resources including provider referral, study flyers, radio and newspaper advertisement, health fairs, social media, and registries maintained by the Pain Research & Intervention Center of Excellence and the Clinical and Translational Science Institute at the University of Florida (from participants who provided consent to be contacted for future research).

#### Inclusion criteria

Given the comorbidity with other pain conditions (Gore et al., 2012; Schneider et al., 2007) and to generalize results more broadly, participants with other musculoskeletal pain conditions were eligible as long as low back pain was identified as the primary pain concern. Additionally, participants were included if they met the following criteria:

- Age ≥50 years
- Endorsement of pain in the lower back region (i.e. space between the lower posterior margin of the rib cage and the horizontal gluteal fold (Deyo et al., 2015)
- Back pain reported of moderate or severe intensity (rating of ≥3 on a 0–10 numeric rating scale)
- Back pain occurring on at minimum half of the days in the past 6 months (Deyo et al., 2015)
- Back pain reported of moderate or severe interference with daily activities (rating of ≥3 on a 0–10 numeric rating scale)

#### Exclusion criteria

To ensure that participants were not at increased risk for discomfort or harm and to protect the integrity of the data due to medical comorbidities that may impact study findings, participants were excluded for the following:

- Current participation in another psychological treatment for chronic pain
- Severe psychiatric illness not adequately controlled by medication (e.g. schizophrenia, bipolar disorder) or other conditions anticipated to impair intervention engagement (e.g. substance abuse/dependence)
- Presence of chronic, malignant pain (e.g. cancer) or systemic inflammatory disease (e.g. rheumatoid arthritis)
- Significant cognitive impairment
- Inability to read and write English
- Back surgery within the past 6 months or future surgeries scheduled within the study time-frame

### Intervention protocol

Intervention content was adapted from positive psychology concepts focused primarily on increasing positive affect, pain acceptance, hope, and self-efficacy; traditional cognitive–behavioral therapy for pain (CBT); and acceptance and commitment therapy (ACT). A full description of the activities and content in the intervention program is summarized in Lysne et al. (2021) (Lysne et al., 2021). Each skill was paired with a home practice exercise, with activities tailored to suit the study population. For instance, adaptations were made to ensure age-appropriate content (e.g. using examples of pleasant activities commonly enjoyed by older adults) and incorporating scenarios specific to chronic pain (e.g. examples denoting challenges related to cLBP). Additionally, written materials featured larger font sizes to accommodate participants with possible visual impairments and images or illustrations depicted older adults to enhance relevance. See Table 1 for a description of the intervention content and home activities, including a list of behavior change techniques underlying each module, as proposed by Michie et al. (2013).

**Table 1.** Intervention session content.

Session	Content	Home Activity	Behavior Change Techniques Underlying Content/Skills
Week 1	BPS model of pain and importance of promoting resilience	Completion of the *Values in Action Survey of Character Strengths* and recording of personal strengths	Social support (emotional); Information about health and emotional consequences; Valued self-identity
Week 2	Gratitude, pleasant activities, and activity pacing	Engage in 3 pleasant activities and complete daily gratitude practice	Problem-solving; Action planning; Self-monitoring of behavior and outcome of behavior; Social support (emotional); Instruction on how to perform a behavior; Information about health and emotional consequences; Behavioral practice/rehearsal; Social reward
Week 3	Values-based activity and mindfulness	Engage in 3 days of mindfulness practice and complete a valued living assessment	Discrepancy between current behavior and goal; Self-monitoring of behavior and outcome of behavior; Social support (emotional); Instruction on how to perform a behavior; Information about emotional consequences; Behavioral practice/rehearsal; Social reward; Conserving mental resources; Valued self-identity
Week 4	Hopeful thinking and valued goal pursuit	Select a personal goal to achieve and identify areas that inspire hope	Goal setting (behavior); Problem solving; Goal setting (outcome); Action planning; Self-monitoring of behavior and outcome of behavior; Social support (emotional); Instruction on how to perform a behavior; Social reward; Mental rehearsal of successful performance; Focus on past success; Self-talk
Week 5	Positive reappraisal of stressful events and savoring	Positively reframe negative thoughts and record weekly positive experiences	Self-monitoring of behavior; Social support (emotional); Instruction on how to perform a behavior; Information about emotional consequences; Behavioral practice/rehearsal; Social reward; Reduce negative emotions; Framing/reframing; Self-talk
Week 6	Self-efficacy for back pain management	Daily practice of diaphragmatic breathing exercise	Social support (emotional); Instruction on how to perform a behavior; Information about health and emotional consequences; Prompts/cues; Behavioral practice/rehearsal; Social reward; Reduce negative emotions; Verbal persuasion about capability; Self-talk; Focus on past success
Week 7	Review and maintenance of skills	Encouragement of continued practice of positive skills.	Problem solving; Action planning; Review behavior goals; Social support (emotional); Information about health and emotional consequences; Comparative imagining of future outcomes; Social reward

### Intervention delivery and treatment fidelity

To standardize the application of the intervention and to ensure treatment fidelity, the intervention was manualized and included interventionist and participant workbooks with materials (e.g. meditation CD) and handouts for discussion and home practice. Sessions were administered by Ph.D. level psychologists with backgrounds in clinical psychology. Interventionists were trained and supervised by the study investigators (EJB, KTS), each of whom has expertise in delivering psychological interventions for individuals with chronic pain. Interventionists received extensive didactic and experiential training from the study PI prior to delivering the program and to ensure uniform delivery of the intervention.

### Measures

#### Primary outcomes: feasibility and acceptability

##### Recruitment and retention

To assess feasibility of the intervention, data were collected on the number of participants enrolled in study procedures and the number of intervention sessions completed, including participant retention at the final intervention session.

##### Home activities evaluation

Participants completed a weekly 7-item questionnaire that was developed based upon items derived from Kazantzis and colleagues (Kazantzis et al., 2004) for the purposes of assessing the utility of the home activities. Items were rated on a 7-point Likert scale ranging from 0 (not at all/none) to 6 (extremely/all). Three questions address feasibility which were used for the analysis (i.e. ‘How much of the home activities were you able to do,’ ‘How well did you understand the reason for doing the home activities [e.g. how the skills could be used to manage pain],’ and ‘How reasonable did you find the time/effort needed to complete the home activities’). A mean score was computed across intervention sessions, with higher scores indicating greater feasibility in home activity completion (current sample: *α* = 0.67).

##### Intervention credibility and expectancy

As a measure of treatment expectancy, questions were developed from Borkovek and Nau (1972) and administered during Visit 1 to specifically address credibility of the intervention for pain management (Supplementary File 1). Items were rated on an 11-point Likert scale and addressed reasonableness of the intervention, willingness to undergo treatment, and confidence in the program. An additional item queried on the expected percentage improvement in pain symptoms that was not used in the scoring. A mean score was calculated for the questionnaire. Higher scores were indicative of greater treatment credibility (current sample: *α *= 0.87).

##### Session-level engagement

Since no existing measures were available and validated in older adults at the time of intervention development, treatment engagement questions were specifically created for this study to assess participants’ effort exerted during group activities, completion of homework, and engagement in group discussions (Supplementary File 2). This questionnaire consisted of 6 items rated on a 9-point Likert scale ranging from 0 (none) to 8 (a lot) and was completed by each participant at the end of every session to assess their perceptions of engagement in the session content and usefulness of the home activities. A mean score was computed across sessions, with higher scores indicating greater participant engagement (mean α across sessions = 0.89).

##### Satisfaction with intervention module content

As an evaluation of the topics relevant to specific intervention content (e.g. pleasant activity scheduling, expressing gratitude, noticing and boosting positive events), participants completed a 33-item study-developed questionnaire at the post-intervention timepoint querying on the ‘usefulness of the information covered in each intervention session and the at-home activities assigned for each session.’ Items were rated on a 5-point Likert scale ranging from 0 (not at all) to 4 (extremely), with mean scores computed (current sample: *α* = 0.98).

##### Global treatment satisfaction

Questionnaire items were administered after the final intervention session to assess global treatment satisfaction (Attkisson & Zwick, 1982). Eight items were rated on a 4-point Likert scale ranging from 1 to 4 and measured aspects related to the quality of the intervention, willingness to recommend the intervention to others, and general satisfaction with the intervention. A mean score was calculated, with higher scores indicating greater satisfaction with the intervention (current sample: *α* = 0.93).

##### Post-intervention skill maintenance

At the 3-month follow-up, participants were asked, ‘How often do you practice any skills you learned during the resilience program? For example, this could include challenging negative thoughts, setting a new SMART goal, practicing mindfulness or relaxation, and engaging in a pleasant activity, among others.’ This item was rated across four response options including ‘daily,’ ‘weekly,’ ‘monthly,’ and ‘never.’

### Secondary outcomes

#### Patient-Reported outcomes measurement information system (PROMIS) pain intensity

The 3-item PROMIS Pain Intensity short form (Cella et al., 2010) evaluates average and worst back pain during the past 7 days, as well as pain at the time of questionnaire completion by providing a 1 (no pain) to 5 (very severe) pain rating. This scale demonstrates adequate reliability and validity in chronic pain populations (Stephan et al., 2021), with acceptable internal consistency observed in the present sample (*α*_T1 _= 0.68, *α*_T2 _= 0.54, *α*_T3 _= 0.71).

#### PROMIS pain interference

The short form of the PROMIS Pain Interference measure (Amtmann et al., 2010) includes 8 questions examining pain-related impairment in social, cognitive, emotional, physical, and recreational activities over the past 7 days. Ratings were made from 1 (not at all) to 5 (very much), with higher scores signifying greater interference from pain. The pain interference scale demonstrates convergent validity with related measures and has high internal consistency (Bartlett et al., 2015). Reliability in the current sample was excellent (*α*_T1 _= 0.93, *α*_T2 _= 0.92, *α*_T3 _= 0.94).

#### PROMIS emotional distress scales

The 8-item short forms of the PROMIS Depression Scale and the PROMIS Anxiety Scale (Pilkonis et al., 2011) assess depressive and anxiety-related symptoms over the past 7 days. Respondents rated the frequency of their experience of each symptom from 1 (never) to 5 (always), with higher scores indicating a greater presence of symptomatology. High internal consistency has been demonstrated for both depression and anxiety scales (Bartlett et al., 2015). In the current sample, Cronbach’s alpha was high to excellent for depression (*α*_T1 _= 0.89, *α*_T2 _= 0.93, *α*_T3 _= 0.94) and anxiety (*α*_T1 _= 0.94, *α*_T2 _= 0.93, *α*_T3 _= 0.93).

#### World Health Organization Quality of Life–Brief (WHOQOL-BREF)

The WHOQOL-BREF (Skevington et al., 2004) scale consists of 26 items assessing quality of life (QoL) over the past week in four domains: physical health (7 items), psychological health (6 items), social relationships (3 items), and environmental health (8 items). The scale also includes 2 items addressing overall QoL (‘How would you rate your overall quality of life’) with score options ranging from 1 (very poor) to 5 (very good) and an item addressing health satisfaction (‘How satisfied are you with your health’) with scores ranging from 1 (very dissatisfied) to 5 (very satisfied). The mean score for each domain was calculated and then multiplied by 4 to create a scaled score ranging from 4 to 20. Higher scores for each domain signify a higher QoL. The WHOQOL-BREF demonstrates satisfactory psychometric properties of reliability and validity (Skevington et al., 2004). Internal reliability in the current sample was good across the four domains: physical health (*α*_T1 _= 0.84, *α*_T2 _= 0.88, *α*_T3 _= 0.83), psychological health (*α*_T1 _= 0.74, *α*_T2 _= 0.80, *α*_T3 _= 0.84), social relationships (*α*_T1 _= 0.78, *α*_T2 _= 0.84, *α*_T3 _= 0.80), environmental health (*α*_T1 _= 0.82, *α*_T2 _= 0.86, *α*_T3 _= 0.86).

### Statistical analysis

All PROMIS measures were transformed into T-scores, with norms based on a United States general population mean of 50 (standard deviation of 10). Continuous measures were summarized as means and standard deviations and categorical measures were summarized as counts and percentages. Pre- to post-intervention change (as well as pre to 3-month follow-up change) in outcomes was reported as mean differences with 95% confidence intervals (95% CI); confidence intervals that do not include 0 provide support for statistically significant change following the intervention. Sensitivity analyses were performed by calculating pre- to post-intervention changes across outcomes separately for patients participating in in-person versus telehealth visits. Analyses were performed in JMP Pro 17 (SAS Institute Inc., Cary NC). All participant interviews were conducted 1:1 with the study coordinator either in person or through PHI Zoom and were recorded and transcribed verbatim. Participant responses were summarized, which included computing the number of positive and negative comments for each participant.

## Results

### Participant characteristics

Characteristics of study participants are shown in Table 2. Fifty percent of the participants were female, and the average age of the sample was 69 years (SD = 7.3, Range = 55–86 years). Participants were mostly white (78%), non-Hispanic (92%), had a college degree or higher (76%), not employed (74%), and were married/partnered (54%). Median back pain duration was 10.0 years (Mean = 16.7, SD = 16.3, Range = 1–60 years).

**Table 2.** Participant characteristics.

Characteristics	M or *N*	SD or %
Age (years)	68.98	7.3
Sex		
Female	25	50.0
Male	25	50.0
Race		
White/Caucasian	39	78.0
Black/African American	10	20.0
Asian	1	2.0
Ethnicity		
Hispanic	4	8.0
Non-Hispanic	46	92.0
Education		
≤High School or GED	2	4.0
Some College	8	16.0
Occupational/Technical/Vocational Program	2	4.0
Two-Year College Degree	6	12.0
Four-Year College Degree	18	36.0
Graduate/Professional Degree	14	28.0
Marital status		
Married/Partnered	27	54.0
Not Married/Partnered	23	46.0
Income		
<$20,000	13	26.0
$20,000-39,999	8	16.0
$40,000-59,999	13	26.0
60,000-99,999	6	12.0
>$99,999	9	18.0
Employment		
Employed Full-Time	3	6.0
Employed Part-Time	8	16.0
Not Employed	38	74.0
Other*	2	4.0
Back Pain Duration, median years (25th-75th%)	10 (4–30)	

Note: *Participants reported employment as ‘self-employed’ and ‘other.’

### Feasibility and acceptability

**Figure 1.**
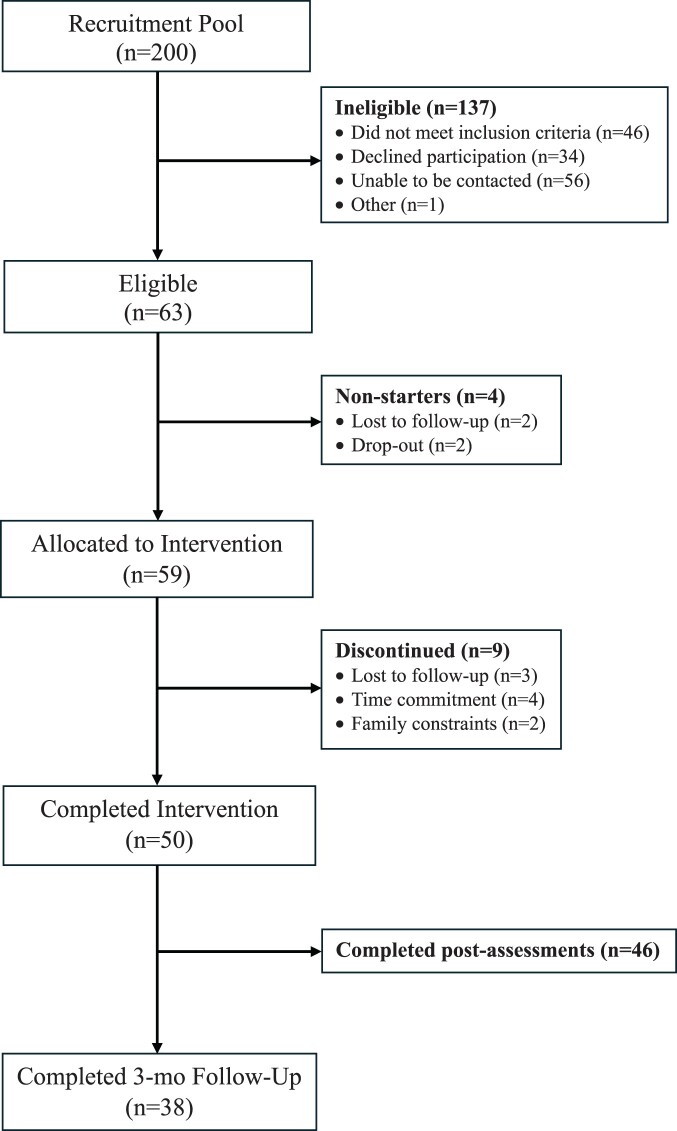
Flowchart of participant recruitment. A flowchart representing participant enrollment and attrition throughout the course of the intervention.

Figure 1 shows the flow of participants through the study. Of the 200 individuals who either expressed interest in the study or who met initial eligibility criteria (via registry status), 63 participants were eligible and enrolled. Of the 63 individuals who were enrolled, 94% of participants (*n* = 59) commenced treatment (i.e. attended intervention session 1). Approximately 85% of participants (*n* = 50) who commenced treatment were retained at the 7-week timepoint. The mean number of sessions completed was 6.86 (SD = 0.35) for completers versus 2.44 (SD = 1.51) for participants who withdrew. Nine participants discontinued after commencing treatment (lost to follow-up, *n* = 3; time commitment, *n* = 4; death in family, *n* = 1; family/caregiving constraints, *n* = 1). Of the 50 participants who were retained through the intervention period, 46 completed all post-intervention assessments including primary and secondary outcome measures (92% retention). The 3-month follow-up assessment was not implemented until the initiation of the second intervention group. Out of the 44 eligible participants, 38 completed the 3-month follow-up questionnaires (86% retention).

Participants rated the home activities as highly feasible (*M* = 4.6/6, SD = 0.88), indicating that they felt capable of completing the home activity content within the weekly timeframe and understood the purpose behind completing the activities. Results also suggest high credibility/expectancy (*M* = 7.7/10, SD = 1.6) and engagement (*M* = 6.3/8, SD = 1.0) in the intervention program. Participants found the weekly session content to be useful (*M* = 2.9/4, SD = 0.78) and global treatment satisfaction was high (*M* = 3.4/4, SD = 0.50), with participants reporting a willingness to recommend the intervention to others and expressing satisfaction with the quality of the intervention. At the 3-month follow-up timepoint, approximately 96% of participants reported continued practice of the skills acquired during the intervention (i.e. daily practice: 41%; weekly practice: 29%; monthly practice: 26%; never: 4%).

Feedback from post-intervention interviews was positive, with several participants reporting improved pain management, increased engagement in previously avoided activities, and a greater focus on self-care and well-being. They found the concepts and home exercises highly beneficial, either by introducing new techniques or reinforcing existing knowledge and skills. Additionally, participants valued the group format for its supportive environment, and noted it was helpful in learning from others’ experiences and feeling reassured that they were not alone in coping with chronic pain. Conversely, participants emphasized a need for clearer instructions for both home activities and questionnaires, and there were varying perceptions on complexity of the material. For instance, while some participants found the content to be simplistic, others suggested simplifying terminology like ‘self-efficacy’ to improve overall understanding. Additionally, participants encountered technological hurdles with Zoom, prompting a preference for in-person sessions to bolster social engagement.

### Preliminary efficacy

Table 3 reports pre- to post-intervention change, and pre- to 3-month follow-up change for outcomes. Both pain intensity (−4.3 [−6.14, −2.54]) and pain interference (−3.3 [−4.83, −1.80]) decreased following the intervention. Additionally, depression (−1.6 [−3.16, −0.04]) and QoL-health satisfaction (0.3 [0.01, 0.55]) improved from pre- to post-intervention. Changes in anxiety, and other QoL measures were minimal.

**Table 3.** Preliminary change in outcomes pre- to post-intervention and pre- to 3-month follow-up.

Measure	Pre-Intervention		Post-Intervention		Change Post-Pre		3-Month		Change 3-Month-Pre	
	Mean	SD	Mean	SD	Mean Dif.	95% CI	Mean	SD	Mean Dif.	95% CI
PR-Pain Intensity	62.10	4.69	57.76	5.73	−4.3	**−6.14, −2.54**	55.37	5.30	−2.1	**−9.29, −5.04**
PR-Pain Interference	59.92	4.37	56.60	5.94	−3.3	**−4.83, −1.80**	55.02	5.78	−5.3	**−6.54, −2.86**
PR-Depression	49.52	7.66	47.92	7.87	−1.6	**−3.16, −0.04**	48.67	8.48	−0.9	−4.61, 0.83
PR-Anxiety	51.87	8.38	52.18	8.47	0.3	−1.39, 2.01	51.61	8.94	−0.1	−2.59, 1.91
WQ-Overall	4.11	0.77	4.11	0.80	0	−0.15, 0.15	4.05	0.90	−0.1	−0.35, 0.09
WQ-Health Satisfaction	3.15	0.94	3.43	1.09	0.3	**0.01, 0.55**	3.42	1.06	0.2	−0.02, 0.44
WQ-Physical Health	13.49	2.90	13.97	2.92	0.5	−0.12, 1.08	14.64	2.62	0.9	**0.11, 1.74**
WQ-Psychological Health	15.07	2.24	15.36	2.24	0.3	−0.21, 0.79	14.86	2.91	−0.2	−0.81, 0.46
WQ-Social Relationships	14.96	3.12	14.28	3.63	−0.7	−1.44, 0.07	13.91	3.80	−1.0	**−1.99, −0.08**
WQ-Environment	16.20	2.33	16.14	2.73	−0.1	−0.44, 0.32	16.30	3.23	0.1	−0.92, 0.80

Note: SD: Standard Deviation; Mean Dif: Mean Difference; 95% CI: 95% confidence intervals; PR: Patient-Reported Outcomes Measurement Information System; WQ: World Health Organization Quality of Life.

At the 3-month follow-up, relative to pre-intervention, pain intensity (−2.1 [−9.29, −5.04]), pain interference (−5.3 [−6.54, −2.86]), and QoL-physical health (0.9 [0.11, 1.74]) improved. However, there were reductions in QoL-social relationships at 3 months (−1.0 [−1.99, −0.08]).

### Sensitivity analysis

Separate analyses were performed for participants having in-person visits (*n* = 10) and those having telehealth visits (*n* = 36), for change from pre- to post-intervention (Table 4). Pain interference improved for participants in both in-person (−2.5 [−4.55, −0.43]) and telehealth (−3.6 [−5.44, −1.66]) intervention visits. When compared to the full sample, telehealth visits showed similar improvements in pain intensity (−5.4 [−7.44, −3.45]), depressive symptoms (−2.4 [−4.27, −0.63]), and QoL-health satisfaction (0.3 [0.01, 0.61]); however, these effects were minimized for in-person visits. While there were no observed changes in psychological health for the full sample, participants receiving telehealth visits exhibited improvements (0.6 [0.01, 1.10]).

**Table 4.** Preliminary change in outcomes pre- to post-intervention, stratified by in-person (*n* = 10) vs telehealth visits (*n* = 36).

Measure	Visit Type	Pre-Intervention		Post-Intervention		Change Post-Pre	
		Mean	SD	Mean	SD	Mean Dif.	95% CI
PR-Pain Intensity	In-Person	60.07	4.28	59.71	6.06	−0.4	−4.05, 3.33
	Telehealth	62.66	4.71	57.21	5.61	−5.4	**−7.44, −3.45**
PR-Pain Interference	In-Person	58.85	5.28	56.36	7.41	−2.5	**−4.55, −0.43**
	Telehealth	60.21	4.13	56.66	5.59	−3.6	**−5.44, −1.66**
PR-Depression	In-Person	46.90	8.23	48.09	7.83	1.2	−1.63, 4.01
	Telehealth	50.32	7.41	47.87	7.99	−2.4	**−4.27, −0.63**
PR-Anxiety	In-Person	51.74	6.65	53.05	8.70	1.3	−2.60, 5.23
	Telehealth	51.92	8.93	51.92	8.51	0	−1.97, 1.97
WQ-Overall	In-Person	3.60	0.84	3.60	0.84	0	−0.34, 0.34
	Telehealth	4.25	0.69	4.25	0.73	0	−0.18, 0.18
WQ-Health Satisfaction	In-Person	2.80	0.92	3.00	1.41	0.2	−0.54, 0.94
	Telehealth	3.25	0.94	3.56	0.97	0.3	**0.01, 0.61**
WQ-Physical Health	In-Person	12.57	3.27	12.74	3.90	0.2	−0.58, 0.92
	Telehealth	13.75	2.78	14.31	2.54	0.6	−0.18, 1.32
WQ-Psychological Health	In-Person	14.73	2.07	14.07	2.30	−0.7	−1.81, 0.48
	Telehealth	15.17	2.30	15.72	2.12	0.6	**0.01, 1.10**
WQ-Social Relationships	In-Person	14.13	3.57	13.60	4.25	−0.5	−1.90, 0.83
	Telehealth	15.19	3.00	14.46	3.48	−0.7	−1.64, 0.20
WQ-Environment	In-Person	14.90	3.37	14.30	3.85	−0.6	−1.61, 0.41
	Telehealth	16.56	1.86	16.65	2.13	0.1	−0.32, 0.50

Note: SD, Standard Deviation; Mean Dif, Mean Difference; 95% CI, 95% confidence intervals; PR, Patient-Reported Outcomes Measurement Information System; WQ, World Health Organization Quality of Life.

## Discussion

In an era marked by rapid growth of the older population, there is an increasing interest in interventions that augment resilience and successful aging, particularly in the context of pain management. Overall, the current study offers support for the feasibility and acceptability of a resilience-promoting positive psychology intervention for older adults with cLBP, as evidenced by high rates of retention and levels of engagement, and satisfaction with the session content. Feedback garnered from post-intervention interviews was largely positive, with participants noting improvements in pain management, increased engagement in previously avoided activities, and a heightened commitment to self-care and well-being. While the current study was not designed to detect intervention efficacy, participants also reported reductions in back pain severity, pain interference, and depressive symptoms, coupled with an improvement in health-related QoL from pre- to post-intervention. Moreover, reductions in pain intensity and interference were sustained at the 3-month follow-up, with approximately 96% of participants continuing to practice the skills acquired during the intervention.

Overall, the findings of this study align with existing research indicating that interventions centered on positive activity engagement can yield benefits in individuals with chronic pain (Braunwalder et al., 2022; Carr et al., 2021; Hassett & Finan, 2016; Hendriks et al., 2020). To promote resilience, we integrated a range of empirically supported strategies from positive psychology (i.e. character strengths, pleasant activity scheduling, gratitude expression, values-based activity, hopeful thinking, positive reappraisal, savoring), hypothesizing that by targeting positive psychological resources, both physical and psychological functioning would be enhanced, serving as markers of increased resilience. Although the mechanisms driving these effects require further investigation, our findings align with current theoretical frameworks suggesting that positive emotions (e.g. gratitude, hope, positive affect) generated during PPIs foster adaptive thoughts and motivational behaviors; in turn, this promotes the building of resources that optimize physical and psychosocial functioning, thereby enhancing resilience (Fredrickson, 2001; Pressman & Cohen, 2005).

Importantly, our study contributes to a growing body of research supporting the efficacy of multi-component PPIs in chronic pain management, with small to large effects reported in mood, physical and psychosocial function, clinical pain severity, and overall well-being (Boselie et al., 2018; Flink et al., 2015; Hausmann et al., 2014, 2017; Janevic et al., 2022; Müller et al., 2016; Müller et al., 2022; Ong et al., 2023; Peters et al., 2017). However, investigations focusing on older adults are limited, as evidenced by only two randomized-controlled trials conducted with this demographic group. These studies have shown promising results regarding the feasibility and initial efficacy of their interventions. In particular, when comparing patients with fibromyalgia who were randomized to a control group (i.e. daily emotion reporting) versus an online self-guided intervention targeting positive psychology skills (i.e. noticing positive events, savoring, strengths, goal-setting, mindfulness, positive reappraisal, gratitude, kindness), Ong et al. (2023) found that participants in the active intervention exhibited reductions in pain catastrophizing and state negative affect and increases in state positive affect. Similarly, in a community health worker-led PPI (i.e. doing what you love, music as medicine, savoring, life review, gratitude, kindness) for chronic musculoskeletal pain involving telephone sessions and web-based videos, Janevic et al. (2022) found that pain interference, pain self-efficacy, global functioning, and pain severity increased in older adult participants relative to a wait-list control condition (i.e. 75-minute intervention session covering intervention content).

While improvements were observed across several outcome domains in the current study, minimal changes were noted in anxiety and various QoL domains, apart from health satisfaction. It is conceivable that the selected measures for these areas of function might lack sensitivity to detect the intervention's effects, or that the intervention itself may not be effective in reducing anxiety symptoms or addressing the components of QoL assessed in the short term. Notably, there was a decline in social well-being from the post-intervention phase to the 3-month follow-up period. While the reasons for this remain unclear, it is possible that reductions in social functioning were due to regression to the mean. Additionally, given that the intervention was conducted during the COVID-19 pandemic, it is plausible that lockdown conditions may have contributed to subsequent decrements in social functioning post-intervention. However, this speculation requires further investigation. As such, social connectedness has been identified as an important determinant of health and contributor to resilience, positively impacting mental and physical functioning, life expectancy, stress management, and coping (Holt-Lunstad, 2022; Ozbay et al., 2007). Although the ARIAA intervention did not directly address social connectedness during skills-based activities, its group format fostered increased social interaction among participants, which may have mitigated reductions in social functioning during the course of the intervention. Future studies may benefit from including booster sessions to bolster support during the transition period following treatment completion. Additionally, integrating therapeutic strategies that improve positive social interactions and social connectivity (Macfarlane, 2020) may help foster social resilience and improve pain-related outcomes.

### Strengths and limitations

Although these findings are promising and suggest that a resilience-promoting intervention can be used successfully with older adults with cLBP, there are several important limitations that warrant acknowledgment. The absence of a control group hinders direct comparisons, preventing a clear assessment of whether the observed effects of the intervention surpass those attributable to standard care or natural fluctuations in symptomatology (i.e. placebo effect). Though aligning with NIH recommendations for this stage of trial development, the decision to employ a single-arm trial design underscores a limitation in terms of assessing the intervention's comparative effectiveness. Thus, findings should be interpreted with caution. Moreover, the exclusive focus on older adults and limited sociodemographic diversity raises questions about the generalizability of the intervention's outcomes. While evidence supports the feasibility and preliminary efficacy of PPIs within older adults (Janevic et al., 2022; Ong et al., 2023), extending conclusions to other age cohorts or populations with distinct characteristics demands further investigation. Given the feasibility nature of the design, the study was underpowered to detect meaningful changes in outcomes or putative mechanisms underlying treatment effects, thereby highlighting the importance of a larger-scale randomized-controlled trial to enhance statistical robustness. Although retention was high, adherence may have been improved by tailoring the exercises to the participants’ activity interest and treatment goals. We also did not collect work-related measures (e.g. work ability index, fear avoidance beliefs questionnaire) to ascertain pain-specific impairments in work ability, and whether the intervention influenced these effects (e.g. changes in work-related stress due to chronic pain). Lastly, due to the pandemic, intervention groups were delivered both via telehealth and in person, resulting in unequal delivery methods across groups. To address this, a sensitivity analysis was conducted to assess potential differences in outcomes based on the delivery method. Interestingly, improvements in pain intensity, depression, and health satisfaction were less pronounced for participants attending in-person sessions, while psychological well-being increased among those receiving remote sessions. However, it is important to interpret these findings cautiously due to the unequal and small sample sizes. Nevertheless, the study provides evidence supporting the effectiveness of telehealth delivery for managing pain in older adults with cLBP. This approach could enhance scalability and expand access to treatment for older adults who may face challenges accessing in-person care.

Despite these limitations, the current study is bolstered by several key strengths. Although there is no universally accepted protocol for positive psychology treatments, our intervention involved activities with established empirical support. Targeting positive psychology concepts, alongside recognized CBT and ACT, has the potential to enhance the therapeutic effectiveness of current pain management strategies and expand the range of intervention options available to patients, especially amongst older adults where cLBP poses a significant concern. Secondly, our 3-month follow-up period allowed us to assess the sustainability of intervention effects and the feasibility of continued engagement in resilience-promoting activities. Lastly, we implemented a group-based format to cultivate a supportive environment and promote social cohesion among participants within the groups, a strategy which may have been beneficial during the pandemic.

## Conclusion and future directions

This study paves the way for future investigations to build upon and explore the efficacy of resilience-promoting interventions for managing pain in older adults. This is particularly important given that older adults are often underserved in psychotherapy due to barriers such as limited access to care, inadequate accommodation for special needs or age-related challenges (e.g. mobility impairments) that can affect treatment engagement, and ageist attitudes among clinicians that may limit referrals to psychotherapy. Despite these challenges, evidence suggests that older adults are especially motivated to engage in psychotherapy and often prefer it over pharmacological treatments (Raue et al., 2009; Vathke et al., 2023).

Overall, positive psychology exercises are low intensity, highly engaging, cost-effective, and allow for self-administration, thereby holding promise for broader implementation with fewer resource constraints. These exercises also resonate with the preferences of older adults, who tend to prioritize emotionally meaningful experiences, favoring positive over negative stimuli in the context of cognitive processing. This preference is consistent with the ‘positivity effect,’ which is a tendency in older adults to focus more on positive stimuli while downplaying negative information. Research suggests that this shift in focus is driven by the motivation to regulate emotions and optimize emotional well-being. As such, positive psychology exercises complement these motivational changes, fostering positive experiences that can enhance emotional resilience and improve overall quality of life in older adults (Gellert et al., 2014; Reed & Carstensen, 2012). Given the limitations of current pain management approaches for older adults, interventions focused on fostering resilience could play a pivotal role in promoting successful aging and increasing the utilization of therapies that enhance pain management and increase function.

## Supplementary Material

Supplemental Material

Supplemental Material

## Data Availability

Data from the current study are available from the corresponding author upon reasonable request.
