# Supplementary material for: Promoting a foundation of resilience in older adults: pilot trial of a strengths-based positive psychology intervention for chronic low back pain
Source: Health Psychol Behav Med. 2024 Dec 5;12(1):2434711. doi: 10.1080/21642850.2024.2434711 (PMC11622378; doi:10.1080/21642850.2024.2434711)
Supplement: Supplemental Material [file RHPB_A_2434711_SM8669.pdf]

Supplementary File 1. Treatment Expectation Questionnaire

There are several statements below that capture your expectations about the resilience intervention. Please indicate to what extent these statements apply to you personally. There are no right or wrong answers. We are only interested in your current personal thoughts.

1. How reasonable does this program seem to you?	◯ 0 = not at all reasonable<br>◯ 1<br>◯ 2<br>◯ 3<br>◯ 4<br>◯ 5<br>◯ 6<br>◯ 7<br>◯ 8<br>◯ 9<br>◯ 10 = completely reasonable
2. How willing would you be to undergo such a program?	◯ 0 = not at all willing<br>◯ 1<br>◯ 2<br>◯ 3<br>◯ 4<br>◯ 5<br>◯ 6<br>◯ 7<br>◯ 8<br>◯ 9<br>◯ 10 = completely willing
3. How confident would you be in recommending this program to a friend or family member?	◯ 0 = not at all confident<br>◯ 1<br>◯ 2<br>◯ 3<br>◯ 4<br>◯ 5<br>◯ 6<br>◯ 7<br>◯ 8<br>◯ 9<br>◯ 10 = completely confident
4. How confident are you that this program will help you cope with your pain?	◯ 0 = not at all confident<br>◯ 1<br>◯ 2<br>◯ 3<br>◯ 4<br>◯ 5<br>◯ 6<br>◯ 7<br>◯ 8<br>◯ 9<br>◯ 10 = completely confident

5. How confident are you that the program will decrease your pain?	◯ 0 = not at all confident<br>◯ 1<br>◯ 2<br>◯ 3<br>◯ 4<br>◯ 5<br>◯ 6<br>◯ 7<br>◯ 8<br>◯ 9<br>◯ 10 = completely confident
6. How confident are you that the resilience program will eliminate your pain?	◯ 0 = not at all confident<br>◯ 1<br>◯ 2<br>◯ 3<br>◯ 4<br>◯ 5<br>◯ 6<br>◯ 7<br>◯ 8<br>◯ 9<br>◯ 10 = completely confident
7. By the end of the program, how much improvement in your pain symptoms do you think will occur?	◯ 0%<br>◯ 10%<br>◯ 20%<br>◯ 30%<br>◯ 40%<br>◯ 50%<br>◯ 60%<br>◯ 70%<br>◯ 80%<br>◯ 90%<br>◯ 100%
