# Supplementary material for: Promoting a foundation of resilience in older adults: pilot trial of a strengths-based positive psychology intervention for chronic low back pain
Source: Health Psychol Behav Med. 2024 Dec 5;12(1):2434711. doi: 10.1080/21642850.2024.2434711 (PMC11622378; doi:10.1080/21642850.2024.2434711)
Supplement: Supplemental Material [file RHPB_A_2434711_SM8668.pdf]

Supplementary File 2. Treatment Engagement Questionnaire

1. How interested were you in the material presented in today's session?	◯ 0 = none<br>◯ 1<br>◯ 2 = a little<br>◯ 3<br>◯ 4 = somewhat<br>◯ 5<br>◯ 6 = much<br>◯ 7<br>◯ 8 = a lot
2. How important to you is the material presented in today's session?	◯ 0 = none<br>◯ 1<br>◯ 2 = a little<br>◯ 3<br>◯ 4 = somewhat<br>◯ 5<br>◯ 6 = much<br>◯ 7<br>◯ 8 = a lot
3. How involved were you in the discussions in today's session?	◯ 0 = none<br>◯ 1<br>◯ 2 = a little<br>◯ 3<br>◯ 4 = somewhat<br>◯ 5<br>◯ 6 = much<br>◯ 7<br>◯ 8 = a lot
4. How comfortable did you feel participating in today's session?	◯ 0 = none<br>◯ 1<br>◯ 2 = a little<br>◯ 3<br>◯ 4 = somewhat<br>◯ 5<br>◯ 6 = much<br>◯ 7<br>◯ 8 = a lot
5. How helpful was the information presented in today's session?	◯ 0 = none<br>◯ 1<br>◯ 2 = a little<br>◯ 3<br>◯ 4 = somewhat<br>◯ 5<br>◯ 6 = much<br>◯ 7<br>◯ 8 = a lot
6. How helpful was the home activity presented in last week's session?	◯ 0 = none<br>◯ 1<br>◯ 2 = a little<br>◯ 3<br>◯ 4 = somewhat<br>◯ 5<br>◯ 6 = much<br>◯ 7<br>◯ 8 = a lot
